# Psychosocial facets of resilience: implications for preventing posttrauma psychopathology, treating trauma survivors, and enhancing community resilience

**DOI:** 10.3402/ejpt.v5.23970

**Published:** 2014-10-01

**Authors:** Brian M. Iacoviello, Dennis S. Charney

**Affiliations:** Department of Psychiatry, Icahn School of Medicine at Mount Sinai, New York, NY, USA

**Keywords:** Resilience, trauma, psychosocial, factors

## Abstract

**Background:**

There is a range of potential responses to stress and trauma. Whereas, on one extreme, some respond to stress and trauma by developing psychiatric disorders (e.g., posttraumatic stress disorder, PTSD), on the other extreme are the ones who exhibit resilience. Resilience is broadly defined as adaptive characteristics of an individual to cope with and recover from adversity.

**Objective:**

Understanding of the factors that promote resilience is warranted and can be obtained by interviewing and learning from particularly resilient individuals as well as empirical research. In this paper, we discuss a constellation of factors comprising cognitive, behavioral, and existential elements that have been identified as contributing to resilience in response to stress or trauma.

**Results:**

The psychosocial factors associated with resilience include optimism, cognitive flexibility, active coping skills, maintaining a supportive social network, attending to one's physical well-being, and embracing a personal moral compass.

**Conclusions:**

These factors can be cultivated even before exposure to traumatic events, or they can be targeted in interventions for individuals recovering from trauma exposure. Currently available interventions for PTSD could be expanded to further address these psychosocial factors in an effort to promote resilience. The cognitive, behavioral, and existential components of psychosocial factors that promote individual resilience can also inform efforts to promote resilience to disaster at the community level.

In the total sense of my life, it's probably been a good experience for me … I wouldn't erase the experience, because I got benefits out of it. I can't erase it. I can't go back and change it … Frankly, it left permanently more good things in my life than it did bad things … Pain creates character … I'm not volunteering to go back, but it's happened. I'll take advantage of the good things … If you can endure pain, you'll learn how to get through hard things. You just become tougher emotionally.

Thomas E. Collins III, former Vietnam prisoner of war

## Trauma comes in many forms, and people's reactions vary

There is a range of potential responses to stress and trauma. On the one hand, retrospective research studies have demonstrated strong associations between the presence of psychiatric disorders and a history of trauma exposure, with the most severe and impairing manifestations being posttraumatic stress disorder (PTSD), depressive disorders, and substance use disorders. On the other hand, it is noteworthy that in most cases, trauma exposure does not lead to psychiatric disorders. For example, one high-quality study based on a large community sample found that the risk of developing PTSD after a traumatic event was approximately 9% (Breslau et al., 1998). Furthermore, some have exhibited a remarkable ability to endure and recover from unfathomable stress, torture, trauma, and disaster. Resilience, as a psychosocial construct, is generally described as adaptive characteristics of an individual to cope with and recover from (and sometimes even thrive after) adversity. Considering the range of stressful and traumatic experiences humans can face, and the range of potential responses, the factors that contribute to resilience compared to psychiatric disorders are an important area of investigation. Understanding these factors can help promote resilience in individuals before they even encounter trauma, can inform psychosocial intervention strategies for treating trauma survivors, and can aid in the development of resilient communities.

Anecdotal evidence from interviews with resilient individuals, and research evidence from studies of trauma and disaster survivors, suggests that a constellation of psychosocial factors contribute to resilience after trauma exposure. These psychosocial factors appear to comprise cognitive, behavioral, and existential components (see Table 1 for a description of the factors and their components). In this context, cognitive components concern people's patterns of thinking or core beliefs, behavioral components concern patterns of action, and existential components concern one's sense of his or her existence, purpose or meaning in the world. This tripartite conceptualization is supported by a factor analysis of the items on a widely used tool used to assess resilience: the Connor–Davidson Resilience Scale (Connor & Davidson, 2003). This factor analysis yielded five factors, which the authors interpreted as: (1) a sense of personal competence and tenacity; (2) tolerance of negative affect and acceptance of the strengthening effects of stress; (3) acceptance of change and cultivating secure relationships; (4) sense of control; and (5) spiritual influences (Connor & Davidson, 2003). Factors 1, 2, and 4 include cognitive components: patterns of thinking and core beliefs that, when confronted with stressful or traumatic situations, lead one to believe they can endure and survive. Factors 1 and 3 include behavioral components: being active and engaged in one's response to stress or traumatic situations, and actively cultivating relationships and social support networks that will enable valuable resources when confronting and recovering from these situations. Factor 5, spiritual influences, represents an existential factor. The literature has also identified neuro-biological factors that appear to influence resilience, including genetic factors, neurochemical systems involved in the stress response and the functioning of specific neural networks (Charney, 2004; Feder, Nestler, & Charney, 2009), although this is beyond the scope of this article. Here we provide a focused discussion of some of the psychosocial factors that have been shown to contribute to resilience, and later we discuss recommendations for cultivating these factors, implications for individual treatment after trauma exposure, and implications for developing resilient communities.

**Table 1:** Cognitive, behavioral, and existential components of psychosocial factors promoting resilience in individuals

	Components		
	
Factor	Cognitive	Behavioral	Existential
Optimism	Maintain positive expectancies for the future.		
Cognitive flexibility	Reappraise, reframe, and assimilate traumatic experiences. Accept stress (trauma) and failure as ingredients for growth.		
Active coping skills (versus passive)	Minimize continued appraisal of threat. Maintain positive self-regard.	Actively seek help and resources.	
Physical health		Physical activity and exercise.	
Social support network		Maintain a social support network.	Not feeling isolated or alone.
Personal moral compass	Adaptive, positive core beliefs.	Altruistic behavior.	Faith/spirituality. Purpose in life.

## Psychosocial factors that promote resilience: cognitive, behavioral, and existential components

### Optimism

One factor that comprises primarily cognitive elements is optimism. Optimism refers to the maintenance of positive expectancies for important future outcomes (Carver, Scheier, & Segerstrom, 2010). Optimism has been conceptualized as a personality dimension, suggesting it is likely more of a trait than a state characteristic, although an individual's degree of optimism can be observed to shift over time or across situations. In research studies, optimism (often in addition to other characteristics) has been associated with psychosocial well-being among long-term breast cancer survivors (Carver et al., 2005), with psychological adjustment during a life transition (Brissette, Scheier, & Carver, 2002), and with lower posttraumatic symptom levels after experiencing a deadly earthquake (Ahmad et al., 2010). As regards resilience in the face of adversity or trauma, maintaining optimism for the future while suffering in the present can buoy one's spirit and provide the stamina to endure. However, optimism alone is not sufficient to foster resilience. The response of James Stockdale, the highest-ranking naval officer held as a prisoner of war in Vietnam and someone known for his resilience to this situation, to the question “Who did not make it out of Vietnam?” reflects this:

Oh, that's easy, the optimists. Oh, they were the ones who said, ‘We're going to be out by Christmas’. And Christmas would come, and Christmas would go. Then they'd say, ‘We're going to be out by Easter’. And Easter would come, and Easter would go. And then Thanksgiving, and then it would be Christmas again. And they died of a broken heart. (Collins, 2014)

### Cognitive flexibility

Stockdale's response illuminates another important factor for resilience, cognitive flexibility, which refers to the ability to reappraise one's perception and experience of a traumatic situation instead of being rigid in one's perception. Reappraisal can also involve an effort to find meaning and positive outcomes as well as acknowledging the negative and painful consequences. Traumatic experiences can be reevaluated, altering the perceived value and meaningfulness of the event. If one can learn to reframe one's thoughts about a traumatic event, assimilate these thoughts into one's memories and beliefs about the event, one may be able to accept and eventually recover. Acceptance and assimilation of a traumatic experience into one's life narrative involves acknowledging that experiences with stress, or even trauma, can provide opportunities for growth. Together, optimism and cognitive flexibility allow an individual to maintain faith that they will prevail or survive regardless of the difficulties at hand, and at the same time, confront and accept the brutal facts of their current reality. This is now known as the Stockdale Paradox (Collins, 2014).

### Active coping skills and maintaining a social support network

Active coping skills, as a factor promoting resilience, incorporates cognitive and behavioral components. Resilient individuals use active rather than passive coping skills; they act and create their own resilience. The cognitive component includes being mindful of one's thoughts about the situations they find themselves in and actively minimizing the appraisal of threat (but not denying threat) so as not to become consumed by fear. At the same time, efforts to create positive statements about oneself and one's situation, and active efforts to seek the help and support of others, comprise the behavioral or action-oriented components. This is also associated with another important factor for promoting resilience, establishing and nurturing a social support network. Very few can “go it alone” and interviews with resilient individuals often yield acknowledgment of invaluable social support. Considerable emotional strength accrues from close relationships with people and even organizations. And the perception of an available “safety net” can enable one to act in one's own interest when confronting or recovering from stressful or traumatic situations. Recent studies of PTSD in veterans returning from wars in Iraq and Afghanistan support this. In one study, PTSD was associated with greater difficulties in relationships, less social support, and poorer social functioning. Importantly, this was not just a consequence of PTSD; less social support from the community and lower availability of secure relationships mediated the association between PTSD and poor social functioning (Tsai, Harpaz-Rotem, Pietrzak, & Southwick, 2012). In another cross-sectional study of predominantly older reserve/National Guard veterans from the same wars, resilient veterans were more likely to be in a relationship and active duty, they scored lower on a measure of psychosocial dysfunction and higher on a measure of post-deployment social support. Being in a relationship, having fewer psychosocial difficulties, and reporting greater perceptions of control and family support were significantly associated with resilience in this cohort (Pietrzak & Southwick, 2011). Moreover, some research has suggested that having social supports in place can influence one's own thinking about oneself and one's worlds in a positive way, protecting against hopelessness and negative psychological outcomes of trauma (Panzarella, Alloy, & Whitehouse, 2006). Taken together, not feeling alone can engender strength to face fear and trauma, and having effective social support can minimize the experience of hopelessness while encouraging adaptive and active coping. This increases the likelihood of resilient outcomes versus psychopathology.

### Physical activity

Physical activity is another factor that is primarily behavioral in nature. Attending to one's own physical well-being before, during, and after facing stress or trauma can promote resilience. Physical exercise improves physical hardiness, which in-and-of-itself can increase the chances of survival from certain traumatic situations. Along with the positive effects on mood and self-esteem that physical exercise confers (Scully, Kremer, Meade, Graham, & Dudgeon, 1998), being mindful of one's physical hardiness during a traumatic situation can contribute to mental fortitude to endure and survive. Improved mood and increased self-esteem after experiencing a traumatic situation could also make establishing and nurturing social and interpersonal relationships easier, which, as noted above, is an important factor in promoting resilience.

### Embracing a personal moral compass

A psychosocial factor that comprises cognitive, behavioral, and existential factors is embracing a personal moral compass. The cognitive component of a personal moral compass involves developing and holding a set of core beliefs that are positive about oneself and one's role in one's world, and that few things can shatter. Studies of the hopelessness theory of depression have shown that hopelessness and depression can stem from maintaining negative core beliefs regarding the stability (enduring over time), globality (permeating different areas of one's life), and internality (regarding one's own personal characteristics) of the negative life events that they encounter (Alloy, Just, & Panzarella, 1997). Conversely, maintaining relatively positive core beliefs results in more adaptive thinking in the face of negative life events, preventing the development of hopelessness, and encouraging resilience. Contributing to one's core beliefs about oneself and one's worlds is the perception that one has of one's own behaviors. So, engaging in positive or altruistic behavior toward others can result in positive core beliefs. Therefore, altruism is an important behavioral component of developing and embracing a personal moral compass, and, in fact, altruism has been strongly associated with resilience in children and adults (Leontopoulou, 2010; Southwick, Vythilingam, & Charney, 2005). Altruistic behavior, or other behavior that confers a sense of sense of community and connectedness, can also contribute to perceived meaning and purpose in life, which represent existential components of embracing a personal moral compass. In a study of psychosocial factors associated with resilience and recovery from the experience of a traumatic event in primary care patients, purpose in life emerged as a key factor associated with both resilience and recovery (Alim et al., 2008). In a study of Pakistani earthquake survivors, purpose in life was associated with lower posttraumatic and depressive symptom levels (Feder et al., 2013).

For many, faith in conjunction with religion or spirituality is also an important existential component of a personal moral compass. Religion and spirituality can provide opportunities for people to ask and gain some understanding of questions about life and personal meaning when facing traumatic situations. This can aid in constructing personal narratives involving healthy perspectives of traumatic situations, and accordingly, contribute to resilience in the face of trauma. Many studies have investigated the relations between religious involvement and mental health. Positive religious coping has been associated with better physical and mental outcomes in response to a range of situations, from disaster survivors (e.g., people affected by large-scale floods; Smith, Pargament, Brant, & Oliver, 2000) to medically ill patients (Pargament, Koenig, Tarakeshwar, & Hahn, 2004). A meta-analysis of the association between religious coping and psychological adjustment to stress, including a total of 13,512 subjects, found that positive religious coping had a moderate positive association with positive psychological adjustment (Ano & Vasconcelles, 2005).

## Cultivating psychosocial factors that promote resilience prior to trauma exposure

Identification of the psychosocial factors that promote resilience begs the question: how can people cultivate these factors as a means of preventing negative outcomes following trauma exposure? The following recommendations map onto the psychosocial factors, identified above, that are often identified in interviews with resilient individuals, and some have been studied and have received empirical research support.

### Find and identify with a resilient role model

Role models can often be found in one's own life. As regards resilient role models, find someone who has experienced adversity, disaster, or trauma. Imitation or modeling can be a powerful mode of learning throughout the lifespan, and finding a resilient role model can be an effective way of cultivating resilience-promoting characteristics via modeling and internalizing the experience of resilience. For example, a role model who has experienced and successfully navigated a traumatic life event can model cognitive flexibility, particularly the experience of acceptance, reappraisal, and assimilation of traumatic experiences. They can become an integral part of a supportive social network, can model active coping skills, and encourage adaptive behavior. They can also model the search for purpose in life and provide spiritual awareness and guidance.

### Establish a supportive social network

As noted above, few can go-it-alone. Having a social support network to learn from before traumatic experiences, and to rely on during and after trauma exposure, can be a valuable resource and can mean the difference between resilient outcomes on the one hand and the development of psychopathology on the other. Close relations with others can contribute to emotional strength. Social support can influence one's perception of oneself and one's worlds (Panzarella et al., 2006), which can contribute to the cognitive components of resilience, particularly maintaining optimism and positive self-regard. A supportive social network can also aid in encouraging active, adaptive coping behavior. People may be more inclined to minimize the appraisal of threat and to act in their own best interest if they perceive a safety net in their social networks, encouraging some of the behavioral components of resilience. Social support networks can include family, friends, co-workers, mentors, and role models, spiritual or religious leaders, and others. Cultivating and nurturing these relationships to form a strong and enduring social support network can be an invaluable means of promoting resilience in the face of trauma.

### Face your fears instead of avoiding them

Oftentimes, our first response to a situation that induces fear or anxiety is to try as hard as we can to avoid the situation and minimize our experience of fear. Fear is an adaptive human experience meant to inform us about potential danger in our environment. So while it is important to listen to this emotion to identify truly dangerous situations, it is also important to acknowledge that avoidance should not be an automatic reaction. Indeed, some psychiatric disorders are conceptualized, in part, in terms of nonacceptance of the experience of fear and maladaptive efforts to avoid fear, anxiety, or uncertainty (e.g., Hayes, Wilson, Gifford, Follette, & Strosahl, 1996). Accepting the experience of fear and anxiety, and pushing oneself to face fears can help promote resilience when experiencing subsequent traumatic experiences. Stress inoculation, involving prior exposure to manageable stressors, has been shown to reduce the behavioral and physiological responses to subsequent stressors (Meichenbaum, 1996). By increasing one's sense of control and mastery of stressful situations, and reducing the amount of anxiety experienced when confronted with a stressful or traumatic situation, one can learn to respond more adaptively. Practice with facing fears provides opportunities for stress inoculation, learning to cope with fear actively and adaptively, and possibly increasing one's self-esteem.

### Attend to your physical well-being

Establishing a regimen of physical exercise and/or activity can have a number of beneficial effects for an individual. Physical exercise contributes to physical hardiness and the practical ability to physically endure or survive certain situations. Physical activity has also been shown to contribute to improved mood and self-esteem (Scully et al., 1998) and an emerging body of multidisciplinary literature has documented the beneficial influence of physical activity on aspects of cognition and brain function (Hillman, Erickson, & Kramer, 2008). A growing number of studies support the idea that physical exercise is a lifestyle factor that might lead to increased physical and mental health throughout life. As regards resilience, physical activity prior to trauma exposure can provide increased self-esteem and optimism about the chances for survival. During and after trauma exposure, physical activity can improve mood and cognitive capacities for emotion regulation, cognitive flexibility, etc.

### Identify, utilize, and foster your particular character strengths

We all have our relative strengths as well as weaknesses. One can make attempts to identify his or her particular character strengths that might contribute to the components of resilience described above. These can include character strengths such extroversion, stress (or fear) tolerance, openness to new experiences, and capacities for emotion regulation, among others. These character strengths can be capitalized on in an effort to cultivate the psychosocial factors that will promote resilience to trauma. For example, extroversion can go a long way to aid in establishing and nurturing a supportive social network. Openness to new experiences and stress or fear tolerance can be capitalized on for stress inoculation and facing one's fears. In addition, we can learn to recognize our character strengths and engage them when confronting and responding to stressful or traumatic situations. Lastly, identifying one's particular character strengths can also help identify relative weaknesses, which one can then work on to further develop and strengthen. Changing in these areas typically involves training regularly and rigorously. Change requires systematic and disciplined activity. Concentrate on training in multiple areas: emotional intelligence, moral integrity, facing fears, physical endurance, etc.

## Implications for psychosocial interventions to promote resilience after trauma

Psychosocial interventions to aid people who have experienced trauma could be tailored to promote resilience by targeting the factors described above. Several psychosocial interventions have already been developed and investigated for individuals who develop PTSD after trauma exposure, comprising elements of cognitive–behavioral psychotherapies. The two interventions that have received the most empirical support are prolonged exposure and cognitive processing therapy (CPT).

Prolonged exposure therapy (PE; Williams, Cahill, & Foa, 2010) is designed to help posttraumatic patients process traumatic events and reduce psychological disturbances. As PTSD is characterized by re-experiencing the traumatic event through intrusive and upsetting memories, nightmares, flashbacks, and emotional and physiological reactions to reminders of the trauma, PTSD patients often try to ward off these intrusive symptoms and avoid any potential trauma reminders which exacerbate their impairment. PE attempts to address this by including two core components: imaginal exposure and *in vivo* exposure. In imaginal exposure, the traumatic memories are revisited and narrated aloud by the patient, enabling them to process the revisiting experience. After repeated experiences with imaginal exposure, *in vivo* exposure involves repeated confrontation with situations and objects that cause distress. Overall, the goal of PE is to promote processing of the trauma memory, and in turn, reduce the distress experienced and avoidance behaviors. In this regard, PE maps onto a behavioral component of resilience, facing one's fears, and the cognitive component of minimizing the continued appraisal of threat. PE has been shown to result in clinically significant improvement in approximately 80% of chronic PTSD patients (Eftekhari, Stines, & Zoellner, 2006), suggesting that these behavioral and components are important for promoting resilience after trauma.

Cognitive processing therapy (CPT; Resick & Schnicke, 1993) conceptualizes PTSD as a disorder of “non-recovery” fueled by erroneous beliefs about the causes and consequences of the traumatic event. These beliefs result in strong negative emotions, and subsequent avoidance of the trauma memory and of situations that trigger reminders. Combined, these prevent adaptive processing of the trauma memory and the emotions emanating from the event. CPT incorporates cognitive techniques to help more accurately appraise these “stuck points” and progress toward recovery, with a primary focus on helping patients to confront, gain an understanding of, and modify the meaning attributed to the traumatic event. The early stage of CPT involves learning to identify automatic thoughts and maladaptive beliefs about oneself and the trauma, and increasing awareness of the relation between one's thoughts and feelings. The next stage of CPT includes formal processing of the trauma, either in writing or verbally with the therapist, to break the pattern of avoidance. The final stage of CPT involves learning cognitive skills to evaluate and modify one's beliefs about oneself and the traumatic events one has experienced, challenging and reframing one's maladaptive conclusions about one's traumatic experience (e.g., “This means that no one can ever be trusted”). CPT has been shown to be effective for a variety of populations and across a variety of trauma types, including military veterans (Monson et al., 2006), victims of sexual assault (Chard, 2005), and refugees (Schulz, Resick, Huber, & Griffin, 2006).

The therapeutic approaches described above represent the most empirically supported, state-of-the-art cognitive–behavioral therapies for recovering from trauma. While they include components that map onto some important factors for promoting resilience, there are also some that are missing and opportunities exist to tailor these approaches to enhance their ability to engender resilience. Table 2 includes examples of the cognitive and behavioral psychotherapeutic techniques, from PE and CPT as well as others, that could be utilized to foster the associated psychosocial resilience factors.

**Table 2:** Psychotherapeutic approaches and techniques to facilitate the psychosocial factors underlying resilience

Factor	Cognitive approaches	Behavioral approaches
Optimism	Modification of core beliefs and expectancies for the future.	
Cognitive flexibility	Practice reappraising, reframing, and assimilating life experiences (traumatic and nontraumatic). Cultivate acceptance for distress, difficulties, etc. Develop mindfulness for thoughts and feelings.	
Active coping skills (versus passive)	Monitor automatic thoughts; minimize continued appraisal of threat; maintain positive self-regard in the face of adversity.	Practice asking for/seeking help and resources. Exposure/inoculation to unwanted/aversive thoughts and feelings. Practice facing one's fears.
Physical health		Behavioral activation: physical activity and exercise.
Social support network		Establish and maintain interpersonal relationships. Connect with a resilient role model.
Personal moral compass	Maintain adaptive, positive core beliefs.	Behavioral activation: altruistic behavior. Engage with faith/spiritual leaders, role models, etc. Engage in activities and goals that yield purpose and meaning for your life.

PE provides opportunities to engage and practice active coping skills: minimizing the continued appraisal of threat and facing one's fears. CPT provides opportunities to engage these active coping skills, and in addition the factors of cognitive flexibility (reappraisal, reframing, and assimilating traumatic experiences; accepting trauma) and embracing a personal moral compass (maintaining adaptive core beliefs; finding meaning in trauma) are also addressed. To enhance these interventions’ ability to promote resilience even further, the remaining psychosocial factors could also be addressed. In particular, efforts to cultivate interpersonal relationships and form a supportive social network are absent in PE and CPT. Some intervention strategies, including brief eclectic psychotherapy for PTSD (Gersons, Carlier, Lamberts, & Van der Kolk, 2000), STAIR narrative therapy (Cloitre et al., 2010), and dialectical behavior therapy (Bohus et al., 2013), which have begun to receive empirical support for the treatment of PTSD, explicitly address enhancing interpersonal effectiveness and/or cultivating a supportive social network in the treatment. Another approach to addressing this could be to pair trauma survivors with a mentor or role model who has successfully navigated his or her own traumatic experience. Including a behavioral activation component (Jacobson, Martell, & Dimidjian, 2001) to these interventions, which has been shown to be effective in the treatment of depressive disorders (Spates, Pagoto, & Kalata, 2006), which often co-occur with PTSD, could also be effective in promoting resilience. Behaviors including physical activity/exercise and encouraging altruistic or other pro-social behaviors might be particularly effective, as they correspond to important psychosocial factors of resilience. Lastly, encouraging spiritual or religious coping as a means of enhancing faith and optimism for the future as well as purpose in life could be included as components of these expanded interventions. Taken together, there exist psychotherapeutic approaches to treating trauma survivors that have been shown to be effective in reducing PTSD symptoms, and there may be opportunities to expand these interventions to include other psychosocial factors to promote resilience even more.

Resilience-focused training programs are being developed that specifically target the psychosocial factors described above. These programs aim to develop resilience by training mindfulness and attention so that one becomes more aware of the present moment as opposed to ruminating on the past and the difficult emotions that engenders. In this way, mindfulness is trained as a means of enhancing emotion regulation. Using purposeful, trained attention, one may decrease the frequency, intensity, and duration of negative thoughts and associated feelings, and bring greater focus on the present moment. In addition, these training programs aim to foster acceptance for the stressful of traumatic event, find meaning in life, develop gratitude, and even address spirituality. Although still in their infancy, such resilience training programs offer the promise of providing an integrated, multimodal approach to promoting resilience, based on the important psychosocial factors described in the literature, and some show promising initial effects on handling daily-life stress in nontraumatized populations (e.g., Rose et al., 2013), while the Comprehensive Soldier Fitness program (Lester, McBride, Bliese, & Adler, 2011), currently being researched in a large-scale study of military soldiers, appears to yield equivocal results (Carr et al., 2013).

Differentiating between interventions aimed at treating the deleterious outcomes of trauma versus primary or secondary prevention interventions, which aim to prevent these outcomes, is an important consideration. The interventions described above are designed to enhance resilience insofar as they seek to reduce the severity of PTSD symptoms experienced after trauma. These interventions are generally provided to individuals who have developed PTSD. However, they could be administered as secondary prevention strategies by administering these treatments to trauma-exposed individuals prior to the emergence of PTSD symptoms. Primary prevention strategies, such as the Comprehensive Soldier Fitness program, are administered to people before they experience a traumatic event, in an effort to provide skills and a foundation for resilience before trauma exposure.

## Implications for developing resilient communities

Unfortunately, community-wide disasters have become more common and costly over the past several decades. Examples of recent large-scale disasters include natural disasters (e.g., hurricanes, floods) as well as man-made disasters (e.g., terrorism), and here too we see a range of responses. Whereas hurricane Katrina in New Orleans and the 2004 Indian Ocean earthquake and tsunami were particularly devastating, in part due to the ineffective early response in the community, responses to the Boston marathon bombing and Superstorm Sandy were indicative of a resilient community response. Community resilience, compared to individual resilience, has been defined as the ability of community members to take meaningful, deliberate, collective action to remedy the impact of a problem, including the ability to interpret the environment, intervene, and move on (Pfefferbaum, Reissman, Pfefferbaum, Klomp, & Gurwitch, 2007). It is the ability to withstand the stress of disaster, recover, and restore functioning, and apply lessons learned from past responses to disaster to better withstand future events.

Norris, Stevens, Pfefferbaum, Wyche, and Pfefferbaum (2008) have elaborated this conceptualization of community resilience as a *process* linking a set of important adaptive capacities in the community to a positive trajectory of functioning and adaptation in constituent populations after a disturbance. This conceptualization highlights the relevant adaptive capacities and identifies the desired outcome as population wellness: a high prevalence of wellness in the community, high and nondisparate levels of mental and behavioral health, role functioning, and quality of life. In an extensive review and synthesis of the literature on community resilience, Norris and colleagues (2008) organize the adaptive capacities of resilient communities into the following clusters: social capital, community competencies, economic development, and information and communication. Social capital refers to the capacities for expected social support, enacted social support, social embeddedness (informal community ties), organizational ties to the community and cooperation, citizen participation (formal community ties), sense of community, and attachment to place. Community competence refers to capacities for community action, critical reflection and problem-solving, flexibility and creativity, collective efficacy and empowerment, and political partnerships. Economic development includes capacities for fairness of risk and vulnerability, level and diversity of economic resources, and equity of resource distribution. Information and communication refers to the capacities for responsible media, skills and infrastructure, trusted sources of information, and narratives of the disaster. Based on this conceptualization, the following practical guidelines for developing these capacities and fostering resilient communities are offered (Norris et al., 2008): (1) communities must develop economic resources, reduce risk and resource inequalities, and attend to the areas of greatest social vulnerability; (2) engage the population meaningfully in every step of the disaster-mitigation process, from preparation to action to recovery; (3) utilize preexisting organizational networks and relationships to rapidly mobilize emergency and ongoing support services for disaster survivors; (4) intervene to support and protect naturally occurring social supports in the aftermath of disaster; and (5) plan, and plan for not having a plan, exercise flexibility and focus on building effective information and communication networks that function in the face of unknowns.

It is noteworthy that the capacities identified as important for promoting community resilience appear to comprise cognitive, behavioral, and existential components, as do the psychosocial factors for individual resilience. Table 3 organizes the capacities identified by Norris and colleagues (2008) into this tripartite model. While Norris’ model appears to include many of the facets of individual resilience, there are some that might also warrant inclusion. For example, this model does not include consideration of the physical health and well-being of constituent populations, which could be addressed by population-wide efforts to promote physical activity for disaster readiness. In addition, community mentors, and faith-based or spirituality organizations, may provide opportunities to organize and support the population during disaster preparation, response, and recovery.

**Table 3:** Cognitive, behavioral and existential components of adaptive capacities promoting resilience in communities

	Components		
	
Adaptive capacity	Cognitive	Behavioral	Existential
Social capital	Perceived social support.	Received social support. Organizational links and cooperation. Citizen participation.	Social embeddedness. Sense of community. Attachment to place.
Community competence	Critical reflection and problem solving skills. Flexibility and creativity.	Community action. Political partnerships.	Collective efficacy/empowerment.
Economic development		Level and diversity of economic resources.	Fairness of risk. Equity of resource distribution.
Information and communication	Trusted sources of information.	Responsible media. Skills and infrastructure.	Narratives.

## Conclusion

People's reactions to stress and trauma can range from resilience, on the one hand, to severe psychopathology, including PTSD, on the other. Understanding of the factors that promote resilience is warranted, and can be obtained by interviewing and learning from particularly resilient individuals as well as empirical research. A constellation of factors, comprising cognitive, behavioral, and existential elements, have been identified in the literature as contributing to resilience in response to stress or trauma. These factors include optimism, cognitive flexibility, active coping skills, maintaining a supportive social network, attending to one's physical well-being and embracing a personal moral compass. These factors can be cultivated even before exposure to traumatic events, or they can be conceptualized and targeted in interventions for individuals recovering from trauma exposure. Currently available interventions for PTSD could be expanded to further address these psychosocial factors in an effort to promote resilience. The cognitive, behavioral, and existential components of psychosocial factors that promote individual resilience can also inform efforts to promote resilience to disaster at the community level.
